# Investigating parents/caregivers financial burden of care for children with non-communicable diseases in Ghana

**DOI:** 10.1186/s12887-015-0504-7

**Published:** 2015-11-16

**Authors:** Aaron A. Abuosi, Francis A. Adzei, John Anarfi, Delali M. Badasu, Deborah Atobrah, Alfred Yawson

**Affiliations:** Department of Public Administration and Health Services Management, University of Ghana Business School, Legon, Ghana; Regional Institute of Population Studies, University of Ghana, Legon, Ghana; Institute of African Studies, University of Ghana, Legon, Ghana; Department of Community Health, University of Ghana Medical School, Korle Bu, Ghana

**Keywords:** Financial burden of care, National health insurance scheme, Cost of hospitalization non-communicable diseases, Children, Ghana

## Abstract

**Background:**

The introduction of the Ghana national health insurance scheme (NHIS) has led to progressive and significant increase in utilization of health services. However, the financial burden of caring for children with non-communicable diseases (NCDs) under the dispensation of the NHIS, especially during hospitalization, is less researched. This paper therefore sought to assess the financial burden parents/caregivers face in caring for children hospitalized with NCDs in Ghana, in the era of the Ghana NHIS.

**Methods:**

We conducted a cross-sectional survey of 225 parents or caregivers of children with NCDS hospitalized in three hospitals. Convenience sampling was used to select those whose children were discharged from hospital after hospitalization. Descriptive statistics such as frequencies and chi-square and logistic regression were used in data analysis. The main outcome variable was financial burden of care, proxied by cost of hospitalization. The independent variable included socio-economic and other indicators such as age, sex, income levels and financial difficulties faced by parents/caregivers.

**Results:**

The study found that over 30 % of parents/caregivers spend more than Gh¢50 (25$) as cost of treatment of children hospitalized with NCDs; and over 40 % of parents/caregivers also face financial difficulties in providing health care to their wards. It was also found that even though many children hospitalized with NCDs have been covered by the NHIS, and that the NHIS indeed, provides significant financial relief to parents in the care of children with NCDs, children who are insured still pay out-of-pocket for health care, in spite of their insurance status. It was also found that there is less support from relatives and friends in the care of children hospitalized with NCDs, thus exacerbating parents/caregivers financial burden of caring for the children.

**Conclusions:**

Even though health insurance has proven to be of significant relief to the financial burden of caring for children with NCDs, parents/caregivers still face significant financial burden in the care of their wards. Stakeholders in health care delivery should therefore ensure that all children with NCDs including those excluded from the NHIS should be covered by NHIS. A special effort focusing on identifying children with NCDs within the lower income groups, especially from rural areas, in order to exempt them from any form of payment for their health care is recommended.

## Background

Universal health coverage has two main components, that is, the extent to which people are covered by the health services that they need, and the degree of financial risk protection they have in using available health services. Do they for example, suffer financially as a result of direct, out-of-pocket payment or high cost of treatment for the health services they need? The World Health Organization (WHO), estimates that over a billion people are unable to use the health services they need; 100 million people are pushed into poverty; while 150 million people face financial hardship because of direct payment for health services at the point of service delivery [1].

The burden of non-communicable diseases is growing and has now become a major problem in the poorest countries. According to WHO, an estimated 36 million deaths, or 63 % of the 57 million deaths that occurred globally in 2008, were due to non-communicable diseases, comprising mainly cardiovascular diseases (48 %), cancers (21 %), chronic respiratory diseases (12 %) and diabetes (3.5 %). In 2008, 80 % of all deaths (29 million) from non-communicable diseases occurred in low- and middle-income countries, and a higher proportion (48 %) of the deaths in the latter countries are premature (under the age of 70) compared to high-income countries (26 %). According to WHO’s projections, the total annual number of deaths from non-communicable diseases will increase to 55 million by 2030, if nothing is done about it [2].

In spite of this situation, the question of whether people have access to the services they need to prevent or control these diseases, and the extent to which they suffer financial catastrophe or impoverishment in obtaining the services they need is less well researched [3, 4]. The situation is even worse in the case of children diagnosed with NCDs. Until late in 2010 when concerns were raised that children risked being systematically excluded from the NCD discourse, the focus was on adults, neglecting the fact that children are not only affected by all the major NCDs, but are the cornerstone to a life course (whole of life) approach to primary prevention and risk factor management [5].

In Ghana, chronic diseases constitute a public health and developmental challenge, requiring the same intellectual and financial commitments afforded to communicable and infectious diseases such as malaria and HIV/AIDS [6]. Even though efforts have been made in recent times in developing a child health policy [7], and a policy on non-communicable diseases [8], a cursory look at these policies would show that they are rather very generic and focusing largely on unhealthy diet and overweight. There are no disease-specific policies on the major NCDs such as cardiovascular diseases (CVDs), cancer, chronic respiratory diseases, diabetes and alcohol [9]. Regarding financial access to the treatment of NCDs, even though some NCDs such as diabetes, hypertension and asthma have been covered by the National Health Insurance Scheme (NHIS), others have been excluded from NHIS coverage. These include cancers other than cervical and breast cancer, heart and brain surgery other than those resulting from accidents, dialysis for chronic renal failure, coupled with diagnostic tests for some of these NCDs such as echocardiography, photography and angiography [10].

Studies have also shown that chronic disease care in Ghana is expensive. The monthly cost of treating conditions like diabetes exceeds the average salary [6]. For example, in 2007, the monthly cost of treating diabetes ranged between $106 and $638; the monthly cost for treating complications of diabetes (e.g. dialysis for end stage renal failure) was $1383 [6]. The minimum daily wage in 2007 was $2; the average monthly salary for a civil servant was $213 [6]. The financial burden of living with chronic disease exacerbates the psychosocial burden. For example, it leads to family disruption and diminished family support. The high cost of treatment of NCDs make them a threat to the lives of those who are diagnosed with them as the average Ghanaian may find it difficult to pay the medical cost for treatment, especially with respect to hospitalized patients. Dror et al. [11] found that hospitalizations were the single most costly component of treatment.

Studies in Ghana suggest that the NHIS eases the financial burden of chronic disease for individuals able to afford the premium payments [12, 13]. The introduction of the NHIS in Ghana has led to progressive and significant increase in utilization of health services. For example, following a three-year trend, per capita out-patient utilization figures increased from 0.81 (2009) to 0.98 (2010) to 1.07 (2011) [14]. That the increase in utilization of health services is being driven in large part by increased enrolment into the NHIS, is evident in the increased number of insured patients as a proportion of total number of out-patients in Ghana’s health facilities: 44.2 % (2009), 55.8 % (2010) and 82.0 % (2011) [14].

With respect to children hospitalized with NCDs two questions arise regarding financial burden of care: first, are many of them enrolled with the NHIS? Secondly, to what extent does membership of NHIS provide financial relief to patients/caregivers whose children are hospitalized with NCDs?

Most studies on NCDs focus on a specific type of non-communicable disease such as diabetes, hypertension or cancer, and hence are not sufficient for understanding the complete burden of care on households from all NCDs [5]. Most studies also focus on adults with NCDs, with less attention to children [5].

This paper therefore seeks to assess the extent to which parents/caregivers of children diagnosed with the major forms of NCDs experience financial burden of caring for children hospitalized with NCDs.

## Overview of literature

Various studies have been conducted in developed and developing countries on the financial burden of NCDs. In the United States of America, Yu et al. [15] investigated whether public insurance provides better financial protection against rising health care costs for families of children with special health care needs, defined to include "those who have or are at increased risk for a chronic physical, developmental, behavioral, or emotional condition and who also require health and related services of a type or amount beyond that required by children generally”. The authors found that families experienced financial burden of care, in spite of public and private insurance. Over 15 % of families with public insurance had financial burden exceeding 10 % of family income compared with 20 % of families with private insurance.

A study in China compared expenditures of members of the New Cooperative Medical Scheme (NCMS), a voluntary health insurance scheme for rural residents, with non-NCMS members in the same areas. Reimbursement from the NCMS was quite low and only 8.67 % of the expenditures of the households seeking care for chronic illnesses was reimbursed in Ningxia and 11.16 % in Shandong. The financial burden on poor households was generally higher than the burden for richer households. Between 14–21 % of families in both provinces suffered from financial catastrophe because of these expenditures, defined as spending more than 40 % of their non-food expenditure on chronic healthcare costs [16].

Another study in China interviewed 671 households enrolled in the Medical Financing Assistance scheme in Wuxi and Qianjiang [17]. These households were all living below the official poverty line. Using multivariate regression analysis, the study found that households where there was at least one member with a chronic illness were 50 % more likely than other households to have incurred debts of greater than 500 RMB (about US$ 60 at that time).

In India, a study found that the odds of incurring catastrophic expenditures on hospitalization were about 160 percent higher for a patient with cancer than the odds of incurring catastrophic expenditure in hospitalization due to a communicable condition. In comparison with cardiovascular disease (CVD), the odds of incurring catastrophic hospital spending were about 30 percent greater compared to communicable conditions that result in hospitalization [18].

In Brazil, a study focused on the richest people in an employer-based insurance scheme in Sao Paolo. Its findings suggest that among the richest, non-communicable diseases were responsible for more than 50 % of the claims for highest spenders in the private health insurance plan [19].

A study in Kenya included 294 rural and 576 urban households in Kilifi district. The authors found that the burden for the poorest quintile was considerably higher than for the richest quintile, reaching 9.6 % of their expenditure in rural areas and 11.8 % of expenditure in urban areas during the recall period of the study [20].

In a study on the health seeking-behaviour and the related household out-of-pocket expenditure for chronic non-communicable diseases in Malawi, Wang et al. [21] found that among those seeking care, 65.8 % incurred out-of-pocket expenditure with an average of USD 1.49 spent on medical treatment and an additional USD 0.50 spent on transport.

A sub-sample of 800 households from the Nouna Health District household survey in Burkina Faso was used to study the incidence of catastrophic health expenditure. The study employed different thresholds of non-food expenditure (from 20–60 % of non-food expenditure) to calculate the incidence of catastrophic health expenditure. Using multivariate regression analysis, it was found that when a household member has a chronic illness, the odds of catastrophic financial consequences associated with paying for health services increased by between 3.3 and 7.8 fold [22].

A rural–urban study of diabetes experiences in Ghana showed that many poor rural men and women with diabetes often relied on financial support from their immediate and distant family members. This dependence on family members who themselves were financially insecure caused family tensions and frictions, which in some cases led to family abandonment and social isolation [23].

Tagoe [24] assessed the burden of non-fatal chronic non-communicable diseases on households in Ghana. The author found that the mean healthcare expenditure for households with respondents currently living with NCDs is 49 % higher than households with healthier respondents. The author concluded that the relatively high direct cost of illness among households with person(s) living with NCDs and the associated high indirect burden of illness places undue stress on households.

## Methods

### Study setting

The study was conducted in three out of the ten regions of Ghana that is, Greater Accra, Ashanti and Volta Regions. The regions were selected by taking into consideration the major ethnic groups so that the socio-economic factors influencing health-seeking behaviours of the various ethnic groups could be captured. Even though there is mixture of ethnic groups in all communities in Ghana, Greater Accra Region is the predominant settlement of the Ga-Adangme ethnic group. Being a coastal region, many of the indigenous people are fisher folk. However, by virtue of being the national capital, Accra also has a lot of people from various parts of Ghana, engaged in trading and industrial activities. Ashanti Region is the predominant settlement of the Akan ethnic group. The region is in the middle portion of Ghana and the indigenous people are engaged in farming cash crops such as cocoa and timber. Much of the minerals extracted in Ghana such as gold and bauxite is in the Ashanti Region. However, Kumasi, is the next cosmopolitan city in Ghana, after Accra. Therefore many people from all over Ghana are engaged in commercial activities in Kumasi. The Greater Accra and Ashanti regions were also selected because Ghana’s leading tertiary hospitals, Korle Bu Teaching Hospital and Komfo Anokye Teaching Hospital, to which most cases of NCDs are referred are located in them. Volta Region is the predominant settlement of the Ewe ethnic group. Many of the indigenous people are engaged in fishing and farming of food crops.

### Study design

We conducted a cross-sectional survey of 225 parents/caregivers of children with NCDS hospitalized in the two teaching hospitals in Accra and Kumasi, and the regional hospital in Ho in the Volta Region. The inclusion criteria were parents/caregivers who were taking care of patients 18 years and below, hospitalized with any type of NCD.

### Sample size determination

The sample size was determined using OpenEpi, Version 3, open source calculator—SSPropor. It was based on the following equation:

$$ Sample\  size\kern0.5em \boldsymbol{n} = \left[\mathbf{DEFF}*\mathbf{Np}\left(\mathbf{1}-\mathbf{p}\right)\right]/\left[\right({\mathbf{d}}^{\mathbf{2}}/{{\mathbf{Z}}^{\mathbf{2}}}_{\mathbf{1}-\boldsymbol{\upalpha} /\mathbf{2}}*\left(\mathbf{N}-\mathbf{1}\right) + \mathbf{p}*\left(\mathbf{1}-\mathbf{p}\right)\Big] $$

where,

- *n* = sample size
- *DEFF* = design effect (used in cluster surveys)
- *N* = population size
- *P* = the hypothesized % frequency of outcome factor in the population
- q = 1-p
- *d* = confidence limits

Since the respondents were in-patients, it was expedient to determine the sample size from the population of in-patients with NCDs in the three hospitals selected, but this was difficult to obtain. However, OpenEpi calculator permits a default population of 1,000,000 as the maximum population size to determine the largest sample size. The hypothesized % frequency of outcome factor in the population, provides an educated guess of the percent of the population with the outcome of interest. In this study the outcome of interest was financial burden of caring for children hospitalized with NCDs. Since respondents were supposed to be contacted personally in the hospitals for interview, the study adopted 85 % as the hypothesized frequency of patients responding to questionnaire on financial burden of care. With the hypothesized frequency of 85 % and confidence limits as ±5, the confidence interval would be 85 % ±5 %, that is, (80 %, 90 %). Based on these specifications, the sample size generated by OpenEpi calculator for the study was 196. However, for convenience and the possibility of non-response, a sample size of 250 was used.

We decided to interview 100 parents/caregivers each in the two teaching hospitals where numbers of NCDs patients are larger, and 50 parents/caregivers in the regional hospital. However, due to lack of patients in the regional hospital, we only interviewed 34 parents/caregivers. For the teaching hospitals, 9 parents/caregivers declined to be interviewed. The final sample size was therefore 225.

### Sampling method and data collection

We employed convenience sampling to select parents/caregivers whose children were hospitalized with NCDs over the study period. The exit interview method was used, that is, parents/caregivers of children diagnosed with the various NCDs discharged daily from the hospital were contacted for the interview when they were leaving the hospital. Convenience sampling was considered appropriate because patients were not discharged en masse, and therefore parents/caregivers of any discharged patient who consented was interviewed. On average, approximately 3–5 parents/caregivers were interviewed per day in each of the three hospitals. Patients hospitalized for other types of care, such as communicable diseases and pre-natal care or institutional deliveries, were not included in the sample. Both patients insured with the national health insurance scheme and uninsured patients were included in the study. Data was collected from 12^th^ to 30^th^ January, 2013. We interviewed the parents/caregivers after they had given informed, written consent. Interviews were conducted by field workers recruited and trained by the Regional Institute for Population Studies in the University of Ghana, Legon.

### Questionnaire

The questionnaire included three sections: (1) patient identity, (2) socio-economic characteristics and (3) financing NCDs. After a thorough training of field supervisors and research assistants, the questionnaire was pretested on 10 patients with NCDs in the University of Ghana General Hospital. After the pretesting, the questionnaire were further refined before the actual data collection began.

### Data analysis

Data was analysed with the aid of SPSS software, Version 20. Descriptive statistics such as frequencies were used to describe the distribution of socio-economic and demographic variables. Chi-square was employed to describe the association of key variables at the bivariate level, such as the association between age, sex, location, income and insurance status, with perception of level of financial burden of care. Finally, logistic regression was used to assess the effects of the socio-economic, demographic and other factors on financial burden of care. Financial burden of care, that is, whether it is expensive or not, was used as a proxy measure of parents/caregivers financial burden. Treatment cost of 51GH¢ ($25.5) was considered expensive, while cost of GH¢50 ($25) and below was considered less expensive. This cutoff, even though arbitrarily determined, is informed partly by the low income levels of respondents, and the fact that close to 70 % of respondents paid GH¢50 ($25) or below, as cost of hospitalization. Thus, the outcome variable is coded 1 if cost of hospitalization (financial burden of disease) is expensive and as 0 if the cost is not expensive. Logistic regression models make it possible to estimate the probability of parents/caregivers’ financial burden of care, conditional on the independent variables included in the model. This takes the form:

1 $$ \mathrm{logit}{p}_i={\beta}_o+{\beta}_i{X}_i+{\varepsilon}_i $$

Taking the linear form:

2 $$ \ln \left({p}_i/\left[1-{p}_i\right]\right)={\beta}_o+{\beta}_i{X}_i+{\varepsilon}_i $$

Where:

- *p*_*i*_= is the probability that the event occurs to an individual with a given set of
- characteristics, *X*_*i*_
- *β*_*o*_= is the intercept or constant
- *β*_*i*_ = is the vector of coefficients, X
- *p*_*i*_/[1 − *p*_*i*_] = is the odds ratio of parents/caregivers with a given set of characteristics considering cost of treatment to be expensive versus not expensive
- *ε*_*i*_ = the error term in the regression

The independent variables include, age of parent/caregiver, age of child, sex, marital status, region, education, religion, location, income, insurance status, and financial difficulties.

### Ethics

The study was approved by the Institutional Review Board of the Noguchi Memorial Institute for Medical Research (NMIMR) of the University of Ghana (Study no. 014/12-13). All respondents were informed of the research objectives and were asked to take part in the study. Those who agreed were asked to sign a consent form.

## Results

### Socio-demographic and other background characteristics of respondents

Table 1 presents the socio-demographic and other background characteristics of respondents. Some of the items of the questionnaire that were not responded to was mainly due to respondents’ unwillingness to respond to those items. Others were due to analysis based on the variable of interest, such as only insured respondents, especially at the bivariate level. To a lesser degree, some respondents did not complete the questionnaire.

**Table 1:** Socio-demographic and other background characteristics of respondents

Characteristic		Frequency (N)	Percentage (%)
Region (N = 225)	Greater Accra Region	97	43
	Volta Region	34	15
	Ashanti Region	94	42
Parents’ Age in years (N = 221)	≤24	33	15
	25 to 30	49	22
	31 to 40	71	32
	41 to 50	43	20
	≥51	25	11
Child age in years (N = 149)	≤5	70	47
	6 to 10	42	28
	11 to 18	37	25
Sex (N = 225)	Male	56	25
	Female	169	75
Education (N = 220)	No education	17	8
	Primary	26	12
	Middle/JSS/JHS	67	30
	Sec/SHS/Vocational Technical	41	19
	Tertiary	69	31
Marital (N = 222)	Never Married	57	26
	Currently married	147	66
	Separated/divorced/widowed	18	8
Location (N = 204)	Urban	167	82
	Rural	37	18
Religion (N = 205)	Christian	180	88
	Moslem	25	12
Income in GH¢ (N = 166)	No income	20	12
	≤100	43	26
	101-200	19	11
	201-300	29	18
	>300	55	33

Table 1 indicates that 43 % of respondents were from Greater Accra region, 42 % were from the Ashanti region and 15 % were from the Volta region. In terms of age distribution, 15 % respondents were 24 years and below, 22 % were 25 to 30 years, 32 % were between 31 to 40 years, 20 % were between 41 to 50 years, and 11 % were between 51 years and above. With regard to the ages of the children hospitalized with NCDs, 47 % were 5 years and below, 28 % were 6 to 10 years, and 25 % were between 11 to 18 years. Male respondents were about one-quarter of the sample (25 %), and females were three-quarters (75 %). With respect to the educational levels of respondents, 8 % had no education, 12 % had primary education, 30 % had middle/JHS education, 19 % had SHS/vocational/technical education, and 31 % had tertiary education. Majority of respondents were married (66 %). However, 26 % were never married, and 8 % were separated/widowed/divorced. Even though the teaching and regional hospitals are referral health facilities, by virtue of their location in urban areas, majority of respondents (82 %) were urban residents, while 18 % were rural residents. Christian respondents constituted 88 %, whereas Muslims were 12 %. There were no respondents from traditional religion. With respect to the levels of income of respondents, 12 % were not earning any income; 26 % earned GH¢100 ($50) or less; 11 % earned between GH¢101 to 200; 18 % earned between GH¢201 to 300; 33 % earned above GH¢300 a month.

### Nature of disease and cost of hospitalization

Out of 120 respondents, 5 % had their children diagnosed with cancer, 19 % were diagnosed with diabetes, 41 % were with sickle cell, 16 % were with congenital deformities, and 19 % were diagnosed with asthma. There were 134 respondents to the question of cost of hospitalization (financial burden of disease). Majority (69 %) spent GH¢50 (approximately $25) or below, and 31 % spent GH¢51 and above. Majority of respondents (87 %) were insured with the NHIS, and 13 % were uninsured. Concerning source of payment for the treatment of NCDs, 78 % of 173 respondents indicated that their source of payment was through the NHIS, while 22 % indicated that it was from personal/other sources of support.

About 4 in 10 respondents (41 %) indicated that they faced financial difficulties in caring for their children during hospitalization, and 59 % indicated otherwise. Concerning whether parents/caregivers received financial support from relatives and friends for the treatment of their children with NCDs, 22 (13 %) out 163 respondents strongly agree that they received financial support and 31 (19 %) agree. On the other hand, 39 (24 %) disagree and 71 (44 %) strongly disagree that they received financial support. In effect, 2 in 3 respondents disagree that they receive financial support from significant others in the health care of their wards.

### Results of Chi-square analysis

From Chi-square analysis, the following key variables had statistically significant association with financial burden of care for parents/caregivers of children with NCDs: region, religious affiliation, income level, insurance status, and parents/caregivers facing financial difficulties during hospitalization. Respondents from the Volta Region were more likely than those from the other two regions to consider financial burden of care to be high χ^2^ (2, N = 134) =11.16, P < .004). Christians were more likely than Muslims to report a higher burden of care χ^2^ (1, N = 122) =4.50, P < .026). Paradoxically, respondents who earned less, tended to pay more for hospitalization, compared with those who earned GH¢300 ($150) and above χ^2^ (4, N = 128) =11.35, P < .023). Regarding insurance status, only 27 % out of 121 insured respondents spent above GH¢50 ($25) as cost of hospitalization. On the other hand, as many as 69 % out of 13 uninsured respondents spent above GH¢50 ($25) as cost of hospitalization χ^2^ (1, N = 134) = 9.60, *P* < .004), indicating, as expected, that the financial burden of care is higher for the uninsured, compared with the insured. The results of this cross-tabulation however had 1 cell (25 %) with expected count less than 5. Finally, respondents who reported facing financial difficulties during hospitalization were more likely to indicate that financial burden of care was high compared with those who did not χ^2^ (1, N = 124) =13.30, *P* < .001). There were however, no statistically significant relationships with the following variables: sex, age, educational level, marital status and location (rural or urban).

### Results of logistic regression analysis

The variables used in the bivariate analysis using chi-square were the same variables used in the logistic regression analysis. Some variables with statistically significant relationship with financial burden of care for children with NCDs remained significant in the logistic regression analysis. These variables include: region of respondents, income levels, insurance status, and financial difficulties facing parents/caregivers with children having NCDs. However, apart from insurance status and financial difficulty which remained significant, all the other independent variables are not significant predictors of financial burden of care. The logistic regression results are shown in Table 2.

**Table 2:** Logistic regression predicting likelihood of financial burden of care

Independent variable	B	S.E.	df	Sig.	Odds ratio
Region (Ref.: Greater Accra)			2	.072	
Volta Region	1.946	2.041	1	.340	7.001
Ashanti Region	−1.536	.837	1	.067	.215
Age (Ref.: ≤24 years)			4	.742	
25 to 30 years	−1.782	1.628	1	.274	.168
31to 40 years	-.286	1.377	1	.835	.751
41 to 50 years	-.679	1.587	1	.669	.507
≥51 years	.061	1.959	1	.975	1.063
Child age (Ref.: ≤5 years)			2	.242	
6 to 10 years	−1.727	1.058	1	.102	.178
11 to 18 years	−1.528	1.201	1	.203	.217
Sex (Ref.: Male)	.117	.970	1	.904	1.124
Education (Ref.: No education)			4	.280	
Primary Education	1.509	1.906	1	.428	4.524
Middle/JHS Education	1.091	1.739	1	.530	2.977
Secondary/Technical Education	1.065	1.772	1	.548	2.901
Tertiary Education	3.421	1.821	1	.060	30.594
Religion (Ref.: Christian)	−2.284	1.766	1	.196	.102
Marital status (Ref.: Never married)			2	.343	
Married	−1.628	1.144	1	.154	.196
Divorced	−1.954	1.779	1	.272	.142
Residence (Ref.: Urban)	.021	1.338	1	.988	1.021
Insurance (Ref.: Insured)	3.154	1.463	1	.031	23.431
Financial difficulty (Ref.: Yes)	−2.805	.988	1	.005	.061
Income level	.077	.139	1	.579	1.080
Constant	2.256	2.618	1	.389	9.548

Dependent variable = Financial burden of care

The model contains eleven independent variables (region of respondent, age of parent/care giver, child’s age, sex, education, religion, marital status, location, income level, insurance status, and financial difficulties). The overall model containing all the predictors (independent variables) was statistically significant, χ2 (20) = 50.11, p < .001, indicating that the model was able to distinguish between respondents who were facing financial burden of care and those who did not. The model as a whole explained between 44 % (Cox and Snell R square) and 62 % (Nagelkerke R squared) of the variance in perceptions of cost of treatment, and correctly classified 83 % of cases.

The results as shown in the table indicate that, two out of the eleven predictors made statistically significant contribution to the model (insurance status and financial difficulties). The strongest predictor of financial burden of care was insurance status, with an odds ratio of 23.4. This indicates, that respondents who were uninsured were about 23 times more likely than insured respondents to pay higher costs of hospitalization, and thus more likely to experience financial burden of care. With respect to financial difficulty, the regression results show that respondents who did not have financial difficulty in paying the cost of hospitalization were 0.06 times less likely to experience financial burden of care.

## Discussion

Within the context of Ghana, this study has found that the health insurance status of children hospitalized with NCDs provides considerable relief to parents/caregivers, in terms of financial burden of care. Results of the chi-square indicate that parents/caregivers of insured children with NCDs were less likely to pay higher cost of hospitalization (27 %), compared with the uninsured (69 %). Insurance status also emerged as the greatest predictor of financial burden of health care in the regression analysis, with the uninsured more likely to pay higher cost of hospitalization, compared with the uninsured. The findings are consistent with conclusions of previous studies in Ghana that the NHIS eases the financial burden of chronic disease for individuals able to afford the premium payments [12, 13].

Other findings of this study however, suggest that health insurance is necessary, but not sufficient to ease the financial burden of care for parents/caregivers of children hospitalized with NCDs. Even though 87 % of respondents were insured, a lower proportion (78 %) of respondents indicated that the source of payment for the cost of hospitalization was through health insurance, while the remaining 22 % was paid out-of-pocket. Also, even though only 13 % of respondents were uninsured, as high as 41 % of respondents acknowledged that they faced financial difficulties during hospitalization. This proportion includes the insured, implying that they also faced financial difficulties. That health insurance is necessary, but not sufficient to address the financial burden of care of children hospitalized with NCDs is consistent with existing literature in developed and developing countries. In America, Yu et al. [15] found that families experienced financial burden of care for children with special health care needs, including NCDs, in spite of public and private insurance. Over 15 % of families with public insurance had financial burden exceeding 10 % of family income compared with 20 % of families with private insurance. In China Sun et al. [16] found that reimbursement from the NCMS, a voluntary health insurance scheme for rural residents, was quite low as only 8.67 % of the expenditures of the households seeking care for chronic illnesses was reimbursed in Ningxia and 11.16 % in Shandong.

The finding that financial difficulty is predictor of financial burden of care for parents/caregivers of children hospitalized with NCDs, is further confirmed by the fact that over 30 % of parents/caregivers of children hospitalized with NCDs pay more than Gh¢50 ($25) out-of-pocket as cost of treatment. Unfortunately, only 1 in 3 respondents indicate any form of financial support from relatives and friends towards the care of their wards. This confirms a rural–urban study of diabetes experiences in Ghana which found that many poor rural men and women with diabetes often relied on financial support from their immediate and distant family members, but these sources are insecure, and sometimes caused frictions, family abandonment and social isolation [23]. The financial difficulty experienced by parents/caregivers of children with NCDs is further corroborated by Tagoe [24] who assessed the burden of chronic non-communicable diseases on households in Ghana. The author found that the mean healthcare expenditure for households with respondent currently living with NCDs is 49 % higher than households with healthier respondents. The author concluded that the relatively high direct cost of illness among households with person(s) living with NCDs and the associated high indirect burden of illness places undue stress on households. Studies in Ghana’s neighbouring country, Burkina Faso, also found that when a household member has a chronic illness, the odds of catastrophic financial consequences associated with paying for health services increased by between 3.3 and 7.8 fold [22]. Studies in other developing countries also have similar conclusions [18, 20, 23].

## Conclusions

This study assessed the financial burden of parents/caregivers of children hospitalized with NCDs.

The study revealed that many children hospitalized with NCDs have been covered by the Ghana national health insurance scheme, and indeed, health insurance provides significant financial relief to parents/caregivers in the care of their children. Notwithstanding the positive effect of health insurance, parents/caregivers still face considerable financial burden of caring for their children with NCDs during hospitalization. The study found that in spite of insurance, both insured and uninsured parents/caregivers pay significant amounts of money out-of-pocket as cost of hospitalization of their children. The financial burden of most parents/caregivers may therefore be exacerbated by these out-of-pocket payments, as well as by the fact that financial support from relatives and friends for the health care of children with NCDs are minimal.

There is need for government through the ministry of health, as well as other stakeholders in health care delivery, to give serious attention to financial access to health care by people with NCDs, especially children. Like other diseases, all children with NCDs including those excluded from the NHIS should be covered by NHIS. A special effort focusing on identifying children with NCDs within the lower income groups, especially from rural areas, in order to exempt them from any form of payment for their health care is recommended.

## Limitations

This study focused on direct cost of hospitalization as a proxy for financial burden of care of children hospitalized with NCDs. Future studies may examine other indirect costs such as cost of transportation and cost of feeding. Future studies may also identify the nature of expenses, including out-of-pocket payments made by parents/caregivers of children with NCDs, in spite of health insurance. Also, a further study is needed to determine why some children with NCDs are not willing or able to enroll with health insurance, in spite of the evidence of significant financial relief provided by the scheme. Finally, timing of data collection was not factored. Future studies need to examine the time respondents are likely to generate higher income, as in harvest season of farmers, or how regularly salaried workers are paid, whether weekly or monthly, and the effect of these on financial burden of care.

## Abbreviations

CVD: Cardiovascular disease
NCMS: New cooperative medical scheme
OPD: Out-patient department
NHIS: National health insurance scheme
NCDs: Non-communicable diseases
JHS: Junior high school
SHS: Senior high school
